# Antimalarial Properties of Dunnione Derivatives as NQO2 Substrates

**DOI:** 10.3390/molecules24203697

**Published:** 2019-10-15

**Authors:** Monivan Chhour, Agnès Aubouy, Sandra Bourgeade-Delmas, Pierre Pério, Hélène Ternet-Fontebasso, Mahamane Haidara, Gilles Ferry, Françoise Nepveu, Jean A. Boutin, Karine Reybier

**Affiliations:** 1 UMR 152 Pharma-Dev, Université de Toulouse, IRD, UP 31062 Toulouse, France; agnes.aubouy@ird.fr (A.A.); sandra.bourgeade-delmas@ird.fr (S.B.-D.); pierre.perio@univ-tlse3.fr (P.P.); helene.ternet-fontebasso@univ-tlse3.fr (H.T.-F.); mahamanehaidara83@gmail.com (M.H.); francoise.nepveu@fondationpierrefabre.org (F.N.); karine.reybier-vuattoux@univ-tlse3.fr (K.R.); 2 Pôle d’Expertise Biotechnologie, Chimie, Biologie, Institut de Recherches Servier, 125, Chemin de Ronde, 78290 Croissy sur Seine, France; gilles.ferry@servier.com (G.F.); jean.boutin@servier.com (J.A.B.)

**Keywords:** dunnione analogues, *Plasmodium* spp., drug discovery, in vivo efficacy, malaria

## Abstract

Dunnione, a natural product isolated from the leaves of *Streptocarpus dunnii* (Gesneriaceae), acts as a substrate for quinone-reductases that may be associated with its antimalarial properties. Following our exploration of reactive oxygen species-producing compounds such as indolones, as possible new approaches for the research of new ways to treat this parasitosis, we explored derivatives of this natural product and their possible antiplasmodial and antimalarial properties, in vitro and in vivo, respectively. Apart from one compound, all the products tested had weak to moderate antiplasmodial activities, the best IC_50_ value being equal to 0.58 µM. In vivo activities in the murine model were moderate (at a dose of 50 mg/kg/mice, five times higher than the dose of chloroquine). These results encourage further pharmacomodulation steps to improve the targeting of the parasitized red blood cells and antimalarial activities.

## 1. Introduction

The control of malaria, a parasitic disease, is a great challenge for countries in tropical and sub-tropical areas. The parasites *Plasmodium*, mainly *P. falciparum* and *P. vivax*, are responsible for this parasitic disease and are transmitted by the bite of the *Anopheles* mosquitos. Several strategies have been implemented to break the parasite/mosquito/host cycle, but none of them have been able to overcome this disease. Therapeutic strategies adopted in humans have mainly targeted the asexual blood stage of the parasite with artemisinin-based combination therapies recommended by the WHO over the past decades [1]. Understanding the parasite’s resistance to many antimalarial drugs [2], including those in therapeutic combinations [3], requires continuous work to renew therapeutic classes. In previous work, we have shown that indolone-*N*-oxides (INODs), which generate radical intermediates, exhibited strong in vivo activities against *P. falciparum* in a humanized mouse model [4,5,6,7]. The low solubility and rapid metabolism of these compounds in vivo prevented their further development. However, this work made it possible to study their targets within the parasitized red blood cells by demonstrating that INODs are substrates for type 2 quinone-reductase (NQO2) [8]. Moreover, we demonstrated that their activity was linked to their ability to produce reactive oxygen species (ROS) after metabolism by autoxidation of the reduced forms [9,10]. In parallel, the work we have carried out to identify new NQO2 substrates showed that *ortho*-quinones were particularly suitable to produce a futile cycle during which the quinone/hydroquinone/quinone cycle in aerobic conditions produced massive bursts of ROS [11]. Ultimately, we focused on the possibility of using analogues of dunnione, a natural compound [12,13], thought to be a substrate of quinone-reductase 1 (NQO1) [14]. On the one hand, we know that red blood cells do not possess NQO1 [15], and on the other hand, we understand that NQO2 has a superior capacity to handle *ortho*-quinone reduction [11]. Thus, NQO2 might be specifically produced by this pathway, overwhelming the amounts of ROS in infected red blood cells. Furthermore, previous studies linked NQO2 with malaria [16], particularly the reverse pharmacology work of Graves et al. [15] as well as our own observations on indolone-type derivatives [8]. Indeed, Graves et al. showed that red blood cells are deprived of NQO1 while presenting a fair amount of NQO2 [16]. We further reasoned that infected blood red cells would be more sensitive to ROS, as they are already challenged by the presence of the parasite. This outcome could lead to the possibility that the substrate of NQO2, subsequently to their reduction by NQO2, could spontaneously be reoxidized, leading to an indirect NQO2-dependent burst of ROS in already compromised blood red cells.

**Figure 1:**
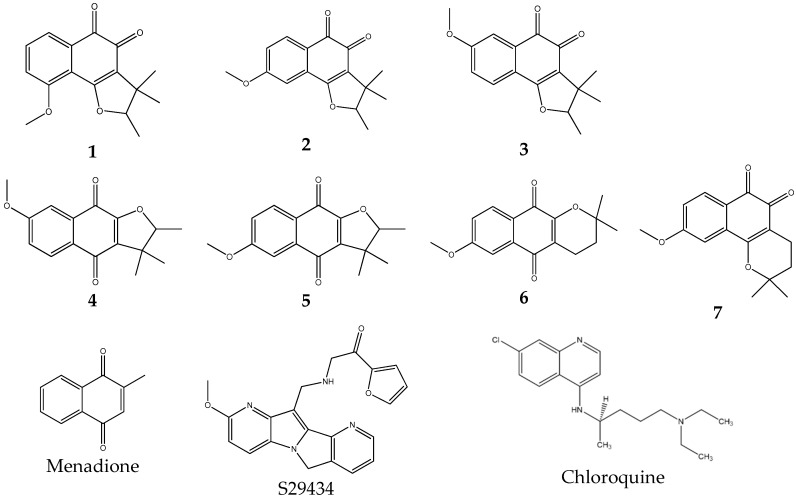
Chemical structures of the compounds tested.

In the present work, we tested three tricyclic compounds and four dunnione derivatives for their antiplasmodial properties (Figure 1: Compounds **1**, **2** and **3**, and Compounds **4**, **5**, **6** and **7**, respectively). These compounds are either *para* or *ortho* quinones. Based on both their ability to inhibit the parasite’s growth in vitro and to produce free radicals, two compounds, **1** and **3**, were selected for in vivo testing on two malaria experimental murine models mimicking uncomplicated malaria (*P. chabaudi chabaudi* infection) and cerebral malaria (*P. berghei* ANKA infection).

## 2. Results and Discussion

### 2.1. Rationale for Selecting NQO2 Substrates

Our previous work related the antimalarial activities of indolone-*N*-oxides to their ability to produce ROS. We showed that the production of ROS was in fact linked to the lack of stability of the product of the quinone reduction reaction. Indeed, the quinone, once reduced, in aerobic conditions, immediately was oxidized back to the original quinone, with the concomitant production of ROS [11]. As indolones [8,10] and many quinones were substrates of the NQO2 enzyme [5], we hypothesized that potent substrates could open new routes to a new class of antimalarial compounds, by way of their instability as reduced quinones (quinols). Bian et al. demonstrated that dunnione, a natural *ortho*-quinone from the Gesneriaceae family, was a substrate of NQO1 and that it had antimalarial properties [14]. Based on this observation, we wondered if other close analogues of dunnione, particularly *ortho*-quinones, could be better candidate(s) than the original compounds as antimalarial agent(s), assuming that NQO2 would better accommodate this class of quinones than NQO1 [11].

The structures of the compounds tested are given in Figure 1.

### 2.2. Rationale for Selecting NQO2 Substrates for Testing In Vivo Antimalarial Properties

As in previous work, we used an indirect assay to characterize the substrates. As seen in Figure 1, some of the quinones used in the present work are *ortho*-quinones. Using the capacity of NQO2 to reduce quinones into hydroquinones or quinols, we incubated the substrate candidates with the enzyme and its co-substrate, a synthetic hydride donor, *N*-benzyldihydronicotinamide. Due to the poor stability of the products of the reaction in aqueous conditions, the product of the reaction, the hydroquinone, was reoxidized almost immediately to the corresponding quinone, leading to massive bursts of reactive oxygen species (ROS) (see below). Thus, using an EPR-based assay, it was possible to evaluate the potency of the process by quantifying those bursts by measuring for the presence of free radicals. The rationale of the process was based on the simple idea that such substrates would generate bursts of ROS, which are particularly toxic for infected red cells [14].

The two-electron reduction of quinones by NQO2 generates unstable hydroquinone, which undergoes autoxidation to the parent molecule if it is not excreted from the cell after a conjugation step. This autoxidation produces superoxide radicals (Equation (1)):

(1) $$Q\overset{\mathrm{NQO}2}{\to }{\mathrm{QH}}_{2}+O_{2}\to {}^{•}\mathrm{QH}+O_{2}^{•-}+H^{+};\;{}^{•}\mathrm{QH}+O_{2}\to Q+O_{2}^{•-}+H^{+}.$$

**Figure 2:**
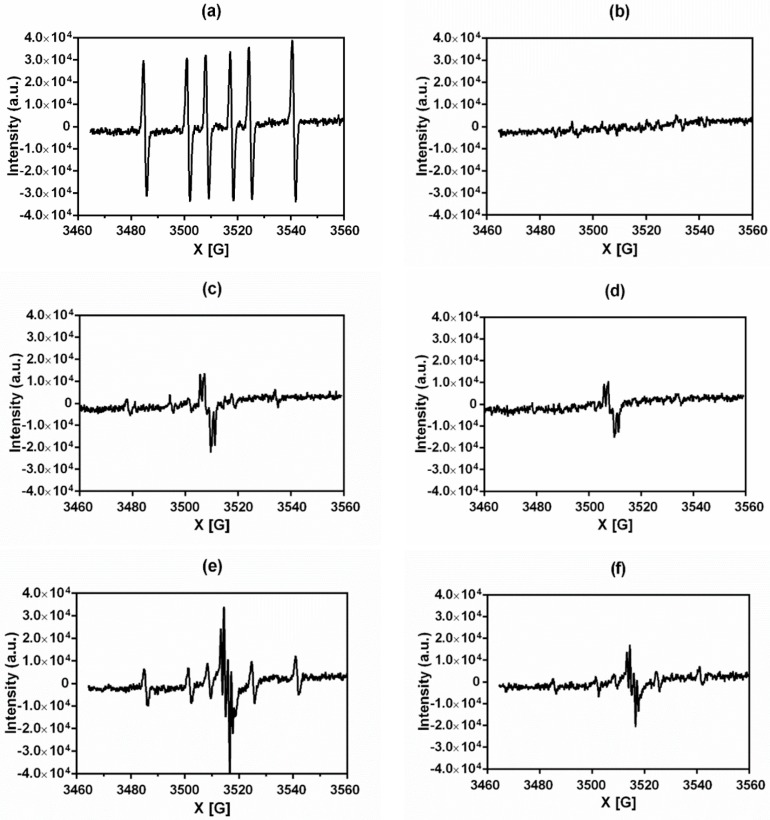
EPR spectra: Free radicals produced by NQO2 metabolism of selected compounds. (**a**) menadione, (**b**) menadione + S29434, (**c**) Compound **1**, (**d**) Compound **1** + S29434, (**e**) Compound **3**, and (**f**) Compound **3** + S29434.

We measured the ability of the selected compounds to produce superoxide radicals during the redox cycle of NQO2 by EPR spectroscopy. The following compounds were used as molecular tools: Menadione (Figure 1) as a reference compound and NQO2 substrate, S29434 as a specific NQO2 inhibitor [17] and dimethyl-pyrroline-*N*-oxide (DMPO) as a spin trap reagent to characterize ROS. Different EPR profiles were obtained, depending on the compound structures as illustrated in Figure 2 for menadione and Compounds **1** and **3**. As already observed [18], the reduction of menadione gives the six-line spectrum characteristic of the DMPO-CH_3_ adduct with hyperfine splitting constants a_N_ = 16.10 G and a_H_^β^ = 23.07 G. The production of methyl radicals originated from the reaction of hydroxyl radicals with DMSO [18]. The dunnione derivatives **1** and **3** presented spectra with marked differences. Indeed, for Compounds **1** and **3**, in addition to the weak six-line spectrum of the DMPO-CH_3_ adduct, a strong multi-lined signal characterized by a hyperfine splitting constant a^H^_H2_ = 3.49 G and a^H^_H1_ = 0.42 G appears. From the literature, this signal can be attributed to the semiquinone radical [19]. This indicates that the semiquinone radical intermediate formed is more stable, thus minimizing the production of ROS (Equation (1)). This result is not the case for menadione. The capacity of the compounds to produce ROS after reduction by NQO2 is schematically summarized in Table 1. Notably, Compounds **1**, **3**, **4** and **6** produced radicals, whereas no radicals were detected for **2**, **5** and **7**. For the first three compounds (**1**, **3** and **4**), the radical production decreased after adding the specific NQO2 inhibitor S29434 [17], confirming that radicals originate in part from the autoxidation of the enzymatically reduced quinone.

The compounds were then tested against the *P. falciparum* strain FcB1 and the results were compared against those with chloroquine. IC_50_ values are given in Table 2. Inhibitory concentrations 50% (IC_50_) of tested compounds were under 2 µM except for Compound **6** (IC_50_ = 19.464 µM). IC_50_ values were greater than the IC_50_ value of the reference antimalarial chloroquine. Compounds **1**, **2**, **3** and **7** were the most active with IC_50_ values ranging from 0.63 to 0.85 µM. The compound toxicity was evaluated using the MTT assay to calculate the cytotoxicity concentration 50% (CC_50_). The Compounds **4** and **5** exhibited CC_50_ values 8–9 fold higher than the IC_50_ value, indicating that these compounds may provide antiplasmodial activity without toxic effects in the host cell. However, the resulting therapeutic indexes are weak for these series. 

Among the four most active compounds in vitro, only **1** and **3** induced a production of ROS after NQO2 metabolism. Therefore, we evaluated the in vivo activity of these two compounds.

### 2.3. NQO2 Substrates As Antimalarian Compounds In Vivo

#### 2.3.1. Acute Toxicity of Compound **1** and Compound **3**

**Figure 3:**
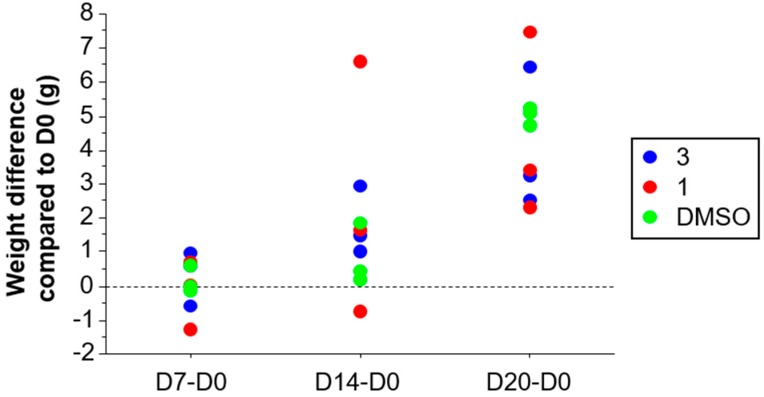
Acute toxicity of Compounds **1** and **3** in healthy Swiss mice. Mice (*n* = 3 per group) were daily treated on D0 to D3 intraperitoneally with 50 mg/kg of Compounds **1** and **3** or their vehicle (50% DMSO). Mice were clinically monitored during 20 days. Results are presented by the difference in weight between the day of follow-up and D0. Weight differences were compared between groups at each point by the Kruskall–Wallis test, *p* > 0.5.

Daily intraperitoneal administration of Compounds **1** and **3** at a dose of 50 mg/kg did not cause mortality or major behavioral changes among experimental animals during the 20 days of administration. Weight variation compared to the vehicle control group was similar between treatment groups at Days 7, 14 and 20 (Figure 3).

#### 2.3.2. Compounds **1** and **3** Treatment Efficacy in the *P. chabaudi chabaudi* Mice Model

**Figure 4:**
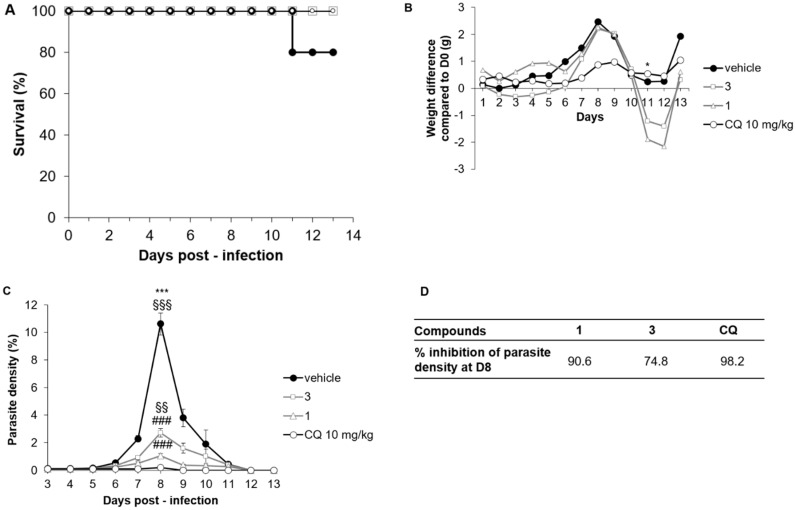
Effect of treatment with Compounds **1** and **3** on survival, parasitemia and weight of *Plasmodium chabaudi chabaudi*-infected mice, compared to chloroquine and vehicle treated animals. Mice (*n* = 5 per group) were infected with 10^6^ *Pcc*/mice at D0 before being daily treated intraperitoneally with **1** and **3** (both at 50 mg/kg), chloroquine (CQ, 10 mg/kg), or their vehicle (50% DMSO) during 4 days (D0 to D3). Mice were clinically monitored during 13 days. (**A**) Survival, (**B**) weight difference compared to D0, (**C**) parasite density (%), and (**D**) % inhibition of parasite density according to treatment. Groups were compared together by Kruskal–Wallis test (* *p* < 0.05, *** *p* = 0.0005), or to CQ by Bonferonni–Dunnett comparison (§§§ *p* < 0.0005, §§ *p* < 0.005), or to DMSO (### *p* < 0.0005).

After *P. chabaudi chabaudi* (*Pcc*) infection in Swiss mice, a non-lethal malaria model, treatment with Compounds **1** and **3** did not cause any death whereas one mouse died in the DMSO group at D11 (Figure 4A). Weight development after *Pcc* infection was similar for the four groups of treatments (DMSO, CQ, **1** and **3**) except at D11, when mice treated with **1** and **3** lost more weight than other groups (Figure 4B). However, weights were again similar at the end of the follow-up period. Interestingly, *Pcc* parasite density was highly reduced after treatment with Compounds **1** and **3** compared to DMSO treatment at D8, the peak of parasitemia (Figure 4C, *p* < 0.0001 for both comparisons **1** and **3** to DMSO). Compared to the CQ treatment, Compound **1** had a similar efficacy at D8 whereas Compound **3** was less effective (Figure 4C, *p* = 0.01). As a result, the inhibition percentages of parasite density at D8 in this model for Compounds **1** and **3** were 74.8% and 90.6%, respectively (Figure 4D), compared to 98.2% for CQ.

#### 2.3.3. Compounds **1** and **3** Treatment Efficacy in a *P. berghei* ANKA Mice Model

**Figure 5:**
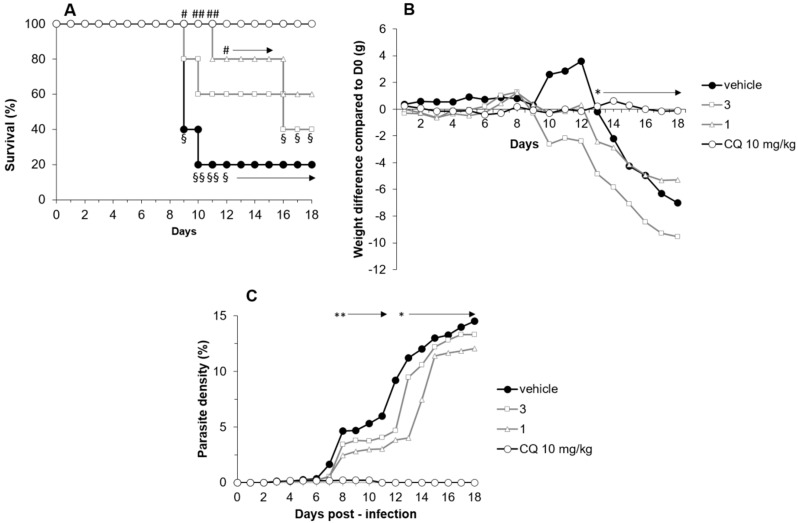
Effect of Compound **1** and **3** treatments on survival, parasitemia and weight of *Plasmodium berghei* ANKA-infected mice, compared to chloroquine and a vehicle. Mice (*n* = 5 per group) were infected with 10^6^ *Pb*A/mice at D0 before being daily treated intraperitoneally with Compounds **1** and **3** (both at 50 mg/kg), chloroquine (CQ, 10 mg/kg), or their vehicle (50% DMSO) during 4 days (D0 to D3). Mice were clinically monitored during 20 days. (**A**) Survival, (**B**) weight difference compared to D0 in g, and (**C**) parasite density (%). Groups were compared all together by a Kruskal–Wallis test (* *p* < 0.05, ** *p* < 0.005), or to CQ by a Bonferonni–Dunnett comparison (§§ *p* < 0.005, § *p* < 0.05), or to DMSO (## *p* < 0.005, # *p* < 0.05).

*P. berghei* ANKA (*Pb*A) infection led to 60% death on D9 and 80% on D10 in mice treated with DMSO. On D10, Compound **1** and **3** treatments allowed survival rates of 80% and 100%, respectively (Figure 5A). The superiority of Compound **1** over DMSO was statistically significant until D15 (*p* = 0.016 on D9, *p* = 0.0025 on D10 and D11, *p* = 0.04 on D12 to D15). Treatment with Compound **1** and **3** did not limit either weight loss or parasitemia during *Pb*A infection, as shown in Figure 5B,C.

## 3. Materials and Methods

### 3.1. Chemicals

Dunnione derivatives (DUNs) were synthesized as described by Bian et al. [14]. They were more than 95% pure, as demonstrated by NMR and mass spectrometry analyses.

The [2-(2-methoxy-5*H*-1,4b,9-triaza(indeno[2,1-a]inden-10-yl)ethyl]-2-furamide (S29434) was prepared as described by Boutin et al. [17], and menadione, Dulbecco’s phosphate buffered saline, Tris-HCL, octyl β-d-glucopyranoside were from Sigma-Aldrich (Saint Quentin Fallavier, France). 5,5′-Dimethyl-1-pyrroline *N*-oxide (DMPO) was from Dojindo (Kumamoto, Japan). *N*-benzyldihydro-nicotinamide (BNAH) was from TCI Europe (Zwijndrecht, Belgium).

### 3.2. EPR Spectroscopy Experiments

The experiments were conducted with purified enzyme NQO2 (20 µg/mL), DMPO (125 mM), tested compounds (125 µM) and BNAH (1 mM). For inhibition assays, we added S29434 (30 µM). All analyses were performed at room temperature. EPR spectra were recorded with a Bruker EMX-8/2.7 (9.86 GHz) with a high-sensitivity cavity (4119/HS 0205) and a gaussmeter (Bruker, Wissembourg, France), at X-band. For analyses, we used a flat quartz cell, FZKI160-5 × 0.3 mm (Magnettech, Berlin, Germany). We performed data processing and spectrum acquisition with WINEPR software (Bruker). Scanning parameters were: Number of scans, 5; magnetic field, 3460–3560 G; sweep width, 100 G; scan rate, 1.2 G/s; modulation amplitude, 1 G; sweep time, 83.88 s; time constant, 40.96 ms; modulation frequency, 100 kHz; and microwave power, 20 mW. The EPR spectra were analyzed as reported previously [18]. The EPR spectrum was presented with the magnetic field on the *x*-axis in gauss (g) and the intensity of the peaks on the *y*-axis in arbitrary units (a.u.).

### 3.3. In Vitro Antiplasmodial Activity

The in vitro anti-malarial activity was investigated using the SYBR Green I-based fluorescence assay. The asexual intra-erythrocytic stage of *P. falciparum* laboratory strain FcB1 (chloroquine-resistant strain) was maintained in RPMI 1640 medium containing l-glutamine 200 µM, Hepes 25 mM and 5% human serum (Etablissement Français du Sang—EFS, Toulouse, France). For anti-malarial drug assays, stock solutions of compounds were diluted serially in RPMI 1640 culture medium to test final concentrations between 0.001 and 1 µg/mL in triplicates in a 96-well plate. The final concentration of DMSO was 0.5%. A suspension of sorbitol-synchronized, infected red blood cells (iRBCs) was adjusted to 1% parasitemia and 2% hematocrit in complete medium and added to the wells. Negative controls were prepared with a suspension of iRBCs and 0.5% DMSO. Chloroquine was used as the positive control. Test plates were incubated at 37 °C for 48 h. Afterwards, 100 μL SYBR Green I fluorescent lysis buffer were added to each well and incubated in a dark place at room temperature for 2 h. Fluorescence data were acquired using a fluorescence plate reader (BMG Fluostar Galaxy Labtech) with excitation and emission wavelengths at 485 nm and 518 nm, respectively. The fluorescence values (after subtraction of the background fluorescence of the non-parasitized RBCs) were plotted against the log of the drug concentration, and analyzed by non-linear regression (sigmoidal dose response/variable slope equation) to yield the IC_50_ (50% inhibitory concentration) that served as a measure of the anti-malarial activity.

### 3.4. Cytotoxic Evaluation on the Hela Cell Line

The evaluation of the tested molecules’ cytotoxicity on the Hela cell line was conducted according to the method of Mosman with slight modifications. Briefly, cells (1 × 10^5^ cells/mL) in 100 µL of complete medium, (MEM supplemented with 10% fetal bovine serum, 2 mM *l*-glutamine and antibiotics (100 U/mL penicillin and 100 µg/mL streptomycin) were seeded into each well of the 96-well plates and incubated at 37 °C and 5% CO_2_. After a 24 h incubation, 100 µL of medium with various product concentrations and appropriate controls were added and the plates were incubated for 72 h at 37 °C and 5% CO_2_. Each plate-well was then microscope-examined to detect possible precipitate formation before the medium was aspirated from the wells. MTT solution (100 µL, 0.5 mg/mL in RPMI) was then added to each well. Cells were incubated for 2 h at 37 °C and 5% CO_2_. After this time, the MTT solution was removed and DMSO (100 µL) was added to dissolve the resulting formazan crystals. Plates were shaken vigorously (300 rpm) for 5 min. The absorbance was measured at 570 nm with a microplate spectrophotometer (Eon BioTek). DMSO was used as blank and doxorubicin (purchased from Sigma Aldrich) as positive control. CC_50_ were calculated by non-linear regression analysis processed on dose response curves, using TableCurve 2D V5 software (San Jose, CA, USA). CC_50_ values represent the mean value calculated from three independent experiments.

### 3.5. Animals and Ethics Statement

In vivo antimalarial tests were realized in the animal house of the PHARMADEV research unit in Toulouse, France. Animal welfare requirements were strictly considered during the experiments and study protocols were reviewed and approved by the Midi-Pyrénées ethic committee for animal experimentation in Toulouse, France. Female healthy Albino Swiss mice aged 6–8 weeks and weighing 26.7–32.9 g were maintained in standard and constant laboratory conditions (23–25 °C and light/dark cycles i.e., 12/12 h) with free access to food and tap water. (Ethic Committee’s permit number APAFIS#5921-2016070118008477 v3).

### 3.6. Acute Oral Toxicity Measurement

Compounds **1** and **3** were diluted in 50% DMSO and tested at 50 mg/kg. Protocols used for acute oral toxicity measurement were previously described [20]. Briefly, mice were randomly divided into three groups of three mice and treated once a day from D0 to D3 by the intraperitoneal route with 200 µL of Compound **1** or Compound **3** at 50 mg/kg, or DMSO 50%. Animals were observed for the first 4 h after treatment to record immediate deaths and once daily for 20 days to record any clinical manifestation of toxicity.

### 3.7. Murine Malaria Models and In Vivo Antimalarial Tests

As previously described [20], the four-days suppressive standard test described by Knight and Peters [21] was used to evaluate antimalarial activity of Compounds **1** and **3**. *P. chabaudi chabaudi* (*Pcc*) and *P. berghei* ANKA (*Pb*A) infections in Swiss mice were used as experimental models for uncomplicated and cerebral malaria, respectively [22,23]. For each model of infection (*Pcc* and *Pb*A), twenty mice were randomly divided into four treatment groups of five mice each: **1**, **3**, chloroquine (positive control) and DMSO 50% (vehicle control) and treated by the intraperitoneal route. On the first day (D0), mice were inoculated intraperitoneally with 0.2 mL of infected blood containing about 1 × 10^6^ parasitized erythrocytes. Two hours after infection, mice were treated by intraperitoneal injection (200 µL/mice) according to their treatment group: Compounds **1** and **3** were both administered at 50 mg/kg, and positive and vehicle control mice received 5 mg/kg of chloroquine and DMSO 50% in distilled water, respectively. All mice groups were treated similarly for four consecutive days (D0–D3). Weight, parasitemia and survival were followed daily. To follow peripheral parasite density, thin blood smears were made daily from tail blood from D3 to D14. Blood smears were fixed with methanol and stained with a fast-acting variation of the May–Grünwald Giemsa staining (RAL 555 kit, RAL diagnostics). Average percentage chemosuppression was calculated at D8 for both infection models as ((A − B)/A) × 100 where A is the average percentage parasitemia in the negative control group and B is the average percentage parasitemia in the test group. Survival was monitored twice daily. The percentage survival was determined over a period of 21 days (D0–D20) and compared between groups.

## 4. Conclusions

Four dunnione derivatives and three tricyclic compounds were tested for their antiplasmodial properties. Apart from Compound **6**, all the products tested have weak to moderate antiplasmodial activities in vitro. The two-electron reduction of these compounds by NQO2 generates the reduced form hydroquinone, which is unstable and rapidly undergoes auto-oxidation to give back the parent compound. This redox cycle results in the generation of the superoxide radical that is EPR-detectable. The presence of the semiquinone radical observed on the EPR spectrum demonstrates the relative stability of this radical that, together with the superoxide radical, contributes to the oxidative burst fatal to the parasite. However, in vivo activities in the murine model are moderate with a dose of 50 mg/kg/day/mice, five times higher than that of chloroquine.

This set of results highlights the value of NQO2 substrates, i.e., molecules capable of undergoing reduction by this enzyme, with the aim to discover new molecular structures with antimalarial activities. In conclusion, it should be pointed out that these molecules have not undergone any pharmacomodulation processes to target the parasitized cell, which will be done in the next steps and should significantly improve antimalarial activities.

## Figures and Tables

**Table 1:** Capacity of the compounds to form reactive oxygen species after metabolism by NQO2.

Compound	ROS after NQO2 Reduction	ROS after Inhibition of NQO2 with S29434
Menadione	+ + + + +	-
1	+ +	+
2	-	-
3	+ + +	+
4	+ + +	+
5	-	-
6	+ + +	+ +
7	-	-
Chloroquine	-	-

**Table 2:** In vitro cytotoxic and antiplasmodial activities of dunnione derivatives (DUNs) on Hela cells and a *Plasmodium falciparum* FcB1 clone, respectively.

Compound	CC_50_ (µM)	IC_50_ (µM)	TI ^1^
Doxorubicin	0.3109 ± 0.478	n.a.	n.a.
Chloroquine	>0.625	0.000164 ± 0.000055	>4000
**1**	2.424 ± 0.905	0.633 ± 0.740	3.83
**2**	1.249 ± 0.160	0.580 ± 0.503	2.15
**3**	1.432 ± 0.314	0.845 ± 0.334	1.70
**4**	11.752 ± 2.196	1.377 ± 0.627	8.53
**5**	14.861 ± 3.226	1.515 ± 0.853	9.81
**6**	>18.362	19.464 ± 7.090	>0.94
**7**	1.665 ± 0.368	0.753 ± 0.484	2.21

CC_50_, 50% cytotoxic concentration; IC_50_, 50% inhibitory concentration. ^1^ Therapeutic Index = CC_50_/IC_50_.
